# Smartwatch Technology for Physical Activity in Older Adults: A Qualitative Study

**DOI:** 10.1093/geroni/igab046.1673

**Published:** 2021-12-17

**Authors:** Mengchi Li, Miranda McPhillips, Sarah Szanton, Jennifer Wenzel, Junxin Li

**Affiliations:** 1 Johns Hopkins University, BALTIMORE, Maryland, United States; 2 University of Pennsylvania, University of Pennsylvania, Pennsylvania, United States; 3 Johns Hopkins University, Baltimore, Maryland, United States; 4 Johns Hopkins University SON, baltimore, Maryland, United States

## Abstract

Older adults’ experiences using smartwatch technology for physical activity (PA) have not been well studied. We studied older adults’ acceptance, capacity, and experience using smartwatches for self-monitoring and promoting PA. We conducted individual interviews using semi-structured interview guides with 15 older adults who participated in two studies. The two studies employed smartwatches in combination with personalized exercise training to promote PA and the interventions were 4 and 24 weeks in length. Interviews were transcribed verbatim. Two researchers conducted inductive content analysis using NVivo V.12 to identify and categorize codes into major themes. Participants reported high overall acceptance, ease of use (i.e., charging, checking steps, reading the screen), and attractive design of smartwatches. Participants’ positive descriptions of their smartwatch experience included: increased activity awareness (step counts and heart rate), improved exercise accountability, and enhanced motivation (response to prompts). Most participants expressed interest in long-term smartwatch use. While participants expressed enjoyment of smartwatch technology for self-monitoring and PA promotion, some reported decreased motivation over time. Participants’ concerns of smartwatch functionalities included short battery life, inaccurate step-recordings, and touchscreen insensitivity. Some also reported failure to troubleshoot smartwatch syncing/pairing problems with smartphones and daily smartwatch charging issues. Smartwatch Bluetooth connectivity and battery life can be improved to increase usability and acceptability among older adults. Future research should explore the role of smartwatches for older adults’ PA with emphasis on behavior change over time.

